# Multi-Element Fingerprinting Combined with Chemometrics for Identification of Seaweeds and Innovative Risk–Benefit Assessment

**DOI:** 10.3390/foods13244159

**Published:** 2024-12-22

**Authors:** Yuansheng Guo, Tiantian Zuo, Shuo Gong, Anzhen Chen, Hongyu Jin, Jing Liu, Qi Wang, Jingjing Liu, Shuai Kang, Ping Li, Feng Wei, Shuangcheng Ma

**Affiliations:** 1 State Key Laboratory of Drug Regulatory Science, National Institutes for Food and Drug Control, Beijing 100050, China; gysheng2022@163.com (Y.G.); zuotiantian@nifdc.org.cn (T.Z.); jhyu@nifdc.org.cn (H.J.); liujing_zsm@126.com (J.L.); sansan8251@sina.com (Q.W.); liujingjing@nifdc.org.cn (J.L.); kangshuai@nifdc.org.cn (S.K.); 2 School of Pharmacy, China Pharmaceutical University, Nanjing 211198, China; liping2004@126.com; 3 School of Integrative Medicine, Anhui University of Chinese Medicine, Hefei 230012, China; gongshuo0221@163.com; 4 NMPA Key Laboratory for Quality Research and Evaluation of Traditional Marine Chinese Medicine, Qingdao Institute for Food and Drug Control, Qingdao 266073, China; 87666368@163.com; 5 Chinese Pharmacopoeia Commission, Beijing 100061, China

**Keywords:** seaweeds, multi-element fingerprinting, chemometrics, species identification, risk–benefit assessment

## Abstract

Seaweeds are one of the major marine foods with high values. The diversity of seaweed species significantly impacts their quality and is closely linked to their purity and safety. For the first time, this study established a model to discriminate seaweed species using a multi-element fingerprinting approach for species identification. Twenty-nine elements derived from seaweeds were analyzed. Chemometrics showed that seaweed samples could be well separated by the established multi-element fingerprints, of which Ag, Mn, Sr, and K were the most important variables for discrimination. Furthermore, the present study proposed an innovative risk–benefit assessment strategy for seaweeds that considers both risks and benefits, developing a novel risk–benefit assessment model from both dietary and medicinal perspectives for the first time. Our innovative strategy was well-conceived to accurately and effectively differentiate seaweeds based on species and scientifically evaluate both benefits and risks associated with seaweeds. This strategy is poised to offer invaluable insights into the sustainable growth of the seaweed sector and to bolster public health initiatives, ensuring a robust and forward-looking approach to both industry and healthcare advancements.

## 1. Introduction

The ocean, covering two-thirds of the Earth’s total surface area, serves as a vast natural treasure trove for humanity and harbors rich biological resources. Its unique ecological environment [1], characterized by high pressure, high salinity, and low temperatures, provides distinct advantages, and it holds immense potential for the development of food and medicine [2,3]. Seaweeds, also known as macroalgae, are a primary source of human food and encompass a diverse array of species. Recent research shows that the major groups of macroalgae include the *Chlorophyta* (green algae), *Rhodophyta* (red algae), *Phaeophyta* (brown algae), *Cyanophyta* (blue–green algae), Diatoms, *Dinophyta* (dinoflagellates), *Cryptophyta* (cryptomonads), and *Chrysophyta* (golden algae) [4,5,6]. Research indicates that globally, more than 100 edible seaweed species are available. In recent years, both the production and consumption of seaweed have been steadily increasing. Seaweeds have become a staple food, particularly in East Asia and the Pacific region [7,8,9]. Previous reports have indicated that in 2022, the global market demand for commercial seaweed exceeded 140 billion yuan (RMB) [10,11]. Seaweed contains abundant proteins, polysaccharides, polyphenols, unsaturated fatty acids, vitamins, minerals, and other substances [12,13]. It is widely used in the functional food, pharmaceutical, biorefinery, dietary supplement, and cosmetic industries [14].

Seaweeds have a wide range of applications not only in functional foods but also in environmental protection and the integration of seaweed with environmental systems, showing tremendous potential. Recent studies indicate that seaweeds can absorb CO_2_ in aquatic ecosystems, providing various ecological benefits such as mitigating coastal pollution and restoring habitats. They also possess strong carbon sequestration capabilities, further helping to combat climate change [15,16]. For example, DeAngelo and colleagues used coupled models of seaweed growth and techno-economic analysis to predict the cost and climate benefits of global seaweed production [17]. Their research suggests that seaweed farming could prevent up to one billion tons of CO_2_ emissions annually. In addition, seaweeds show great potential in wastewater treatment. They can effectively absorb heavy metals and harmful substances from water and are capable of biologically removing nitrogen and phosphorus, thus purifying water quality [18,19,20]. Salim et al. demonstrated the use of green macroalgae as a source for large-pore cellulose monomers, which successfully served as bio-adsorbents for wastewater treatment [21]. Furthermore, due to their high content of polysaccharides and fatty acids, seaweeds are a promising source of bioenergy, making them a sustainable alternative to traditional fuels [22,23,24]. In summary, the multifunctionality and efficiency of seaweeds position them as a crucial resource for modern environmental protection, food security, and sustainable development. Consequently, the demand and production of seaweed are expected to rise steadily, offering great potential for broader applications in the future.

Owing to the diverse varieties of commercial seaweeds, each with varying values and effects, there is confusion in the market regarding the species and misuse of seaweed varieties. Therefore, there is an urgent need to identify the species of different seaweeds. Species identification is essential for determining the sources of food or pharmaceutical varieties. In previous studies, targeted testing techniques, including high-performance liquid chromatography (HPLC) and ultra-performance liquid chromatography coupled with mass spectrometry (UPLC-MS), have been developed as applicable methods for product species identification [25,26]. Although the aforementioned methods offer insights into the species of seaweeds, their chemical composition is susceptible to external factors, making identification challenging. Seaweeds exhibit diverse morphological characteristics that further complicate their identification. Therefore, this research focuses on the species identification of three seaweed varieties, providing a reference for the identification of the species of other seaweeds and food products.

To date, studies utilizing elemental analysis for the authentication of traditional Chinese medicine or food provenance have achieved certain results. However, most previous research has focused on a single or a limited number of elements, which does not fully reflect the elemental profile of the subjects under study. For instance, Zhao et al. explored the feasibility of using multi-element fingerprinting combined with data analysis to distinguish the geographical origin of peanut kernels by analyzing the content of 20 inorganic elements in kernels from different origins [27]. Similarly, Ma et al. demonstrated that the combination of inorganic elemental fingerprinting and multivariate statistical analysis is an effective means of identifying the provenance of Ephedra by analyzing the content of 15 inorganic elements in different samples [28]. While these studies successfully achieved the goal of provenance discrimination using elemental fingerprinting, they had certain limitations in the number of elements studied and lacked research on the risks and benefits of different elements. Building on these studies, we have increased the number of inorganic elements and provided a more comprehensive elemental fingerprint.

Seaweeds are rich in mineral elements, which often exhibit diverse physiological functions owing to their varying contents and specific properties. These minerals are essential substances for the human body, but it is crucial to note that while they offer various health benefits, they also present varying degrees of health risks. When used inappropriately, they may cause health damage to the human body [29,30,31,32].

Therefore, the main objectives of this study were: (1) to establish mineral element fingerprint profiles for three commercial seaweeds and comprehensively characterize the distribution of mineral elements in these seaweeds; (2) to explore the feasibility of seaweed species identification using pattern recognition algorithms based on mineral elements, including both supervised and unsupervised models, aiming to address issues such as confusion in the seaweed market and ambiguity in food authenticity; and (3) to propose a novel risk–benefit assessment strategy for seaweeds, by conducting a comprehensive evaluation of the health benefits and risks associated with mineral elements in seaweeds. This study aimed to clarify the potential risks and benefits of consuming seaweeds for human health, provide valuable insights for the public’s informed consumption, and assist in the formulation of scientifically sound regulatory standards by national authorities.

## 2. Materials and Methods

### 2.1. Seaweed Sample Collection

In total, forty-two batches of seaweed samples were collected from major seaweed-producing areas in China from May in 2022. All the seaweed samples were collected in Shandong Province, Zhejiang Province, and Guangdong Province, and the collection locations and detailed information of each seaweed sample are listed in Table 1, respectively. During the collection process, the research team endeavored to ensure the consistency of the environment and timing for the collection of all seaweed samples.

For processing the seaweed samples, all collected fresh seaweed samples were thoroughly washed and then dried in a constant temperature drying oven at 60 °C for 24 h until they were completely dry. Subsequently, the dried samples were pulverized and sieved through a 50-mesh screen for experimental use.

### 2.2. Chemicals and Reagents

The ultrapure water used for all experiments was supplied by a Mill-Q system (Millipore, Chicago, IL, USA). Multi-element calibration standard solutions (Ag, Al, As, Ba, Be, Cd, Co, Cr, Cs, Cu, Fe, Ga, Li, Mn, Ni, Pb, Rb, Sr, Tl, U, V, and Zn) were obtained from Agilent (Agilent Technologies, Folsom, CA, USA). Single-element standard solutions (K, Ca, Na, Mg, Sb, Sn, and Hg) were obtained from Accu Standard (Accu Standard, New Haven, CT, USA). Tuning solutions containing Li, Y, Ce, Tl, and Co (Part# 5185-5959) and an internal standard solution containing Li, Ge, Rh, In, Tb, Lu, and Bi (Part# 5188-6525) were purchased from Agilent (Agilent Technologies, Folsom, CA, USA). Supra-pure trace metal-grade concentrated nitric acid (HNO_3_, 70.0%) was purchased from Merck (Merck, Munchen, Germany).

### 2.3. Multi-Element Analysis

#### 2.3.1. Seaweed Samples Digestion

Firstly, the PTEE digestion tube was immersed in 10% nitric acid solution for 24 h and then rinsed with ultrapure water to reduce (eliminate) the interference of the apparatus; secondly, the sample powder was weighed 0.3 g, precisely, and placed in the inner tank of the microwave digestion, added 7 mL of nitric acid, installed the device according to the operating procedures, and placed it in the microwave digestion instrument for digestion. At the end of digestion, cool to room temperature, drive the acid, dilute the sample with ultrapure water, and volume to 50 mL, used for elemental analysis. The blank solution was prepared using the same method.

#### 2.3.2. Calibration Procedure

External calibration curves were followed for the quantitative analysis of samples. For ICP-MS, standard solutions were prepared in 5% (*w*/*w*) HNO_3_ by diluting a multi-element standard solution containing 22 elements: Ag, Al, As, Ba, Be, Cd, Co, Cr, Cs, Cu, Fe, Ga, Li, Mn, Ni, Pb, Rb, Sr, Tl, U, V, and Zn, and 7 species single-element standard solutions: K, Ca, Na, Mg, Sb, Sn, and Hg. Under optimized measurement conditions, ten different concentrations (0.10, 1.00, 2.00, 4.00, 10.00, 20.00, 40.00, 100.00, 200.00, and 400.00 μg/L) of Ag, Al, As, Ba, Be, Cd, Co, Cr, Cs, Cu, Fe, Ga, Li, Mn, Ni, Pb, Rb, Sr, Tl, U, V, and Zn, and seven different concentrations (100.00, 500.00, 1000.00, 5000.00, 10,000.00, 50,000.00, and 100,000.00 μg/L) of K, Ca, Na, and Mg, and ten different concentrations (0.10, 1.00, 2.00, 4.00, 10.00, 20.00, 40.00, 100.00, 200.00, and 400.00 μg/L) of Sb and Sn, and nine different concentrations (0.01, 0.02, 0.04, 0.10, 0.20, 0.40, 1.00, 2.00, and 4.00 μg/L) of Hg were measured, and calibration curves were plotted from the limits of detection of the corresponding elements. The internal standards Li, Ge, Bi, Lu, In, and Rh (500 μg/mL) were determined concurrently with multi-elements to ensure stability and accuracy for analysis.

#### 2.3.3. ICP-MS Analysis

Multi-elements (Ag, Al, As, Ba, Be, Cd, Co, Cr, Cs, Cu, Fe, Ga, Li, Mn, Ni, Pb, Rb, Sr, Tl, U, V, Zn, K, Ca, Na, Mg, Sb, Sn, and Hg) were determined using SHIMADZU 2030 inductively coupled with plasma mass spectrometry (ICP-MS, SHIMADZU., Ltd., Shanghai, China). The instrumental parameters were set as follows: The high-frequency plasma power was 1250 W with a detector voltage of -1850 V, and high-purity argon (Ar, 99.999%) was used as the carrier gas. The plasma gas, atomization gas, and collision gas flow rates were 17 L/min, 0.92 mL/min, and 4.5 mL/min, respectively. The peak hopping mode was selected as the acquisition method with three sampling repeats.

#### 2.3.4. Data Processing and Chemometric Analysis

Hierarchical cluster analysis (HCA), principal component analysis (PCA), partial least squares discriminant analysis (PLS-DA), orthogonal partial least squares-discriminant analysis (OPLS-DA) models, and one-way analysis of variance (ANOVA) of elements were used to visualize the distribution trends of algae samples from different species, which were performed using Chempattern 2017 pro software (Chemmind, Beijing, China). Statistical analyses, including means, standard deviation, and range, were calculated in Microsoft Excel 2019 (Microsoft Co, Redmond, WA, USA).

### 2.4. Establishment of a Risk–Benefit Assessment Strategy for Three Species of Seaweeds

#### 2.4.1. Non-Carcinogenic Risk Assessment

For certain elements with authoritative data, the oral reference doses recommended by the U.S. Environmental Protection Agency (US EPA) have been established. Currently, the health-based guidance values (HBGVs) for the following elements are known: Pb, Cd, As, Hg, Cu, Al, Mn, and Ni are 3.5, 1.0, 0.3, 0.6, 500.0, 1000.0, 140.0, and 20.0 μg/(kg·d), respectively. For elements without established health guidance values, their reference intake values are set according to the ’Chinese Dietary Reference Intakes for Residents (2023 Edition). The provisional daily tolerable intakes (PTDIs) for Ca, Fe, Zn, Co, V, and Li are 3110.0, 650.0, 620.0, 0.3, 0.9, and 1.6 μg/(kg·d), respectively. However, K, Na, Mg, and Cr have not yet determined their maximum tolerable intake, so their hazard indices (HI) are not considered in this study. Then, exposure assessment refers to the process of calculating the exposure level (Exp) based on the data of elemental residue in TCMs and foods during the risk–benefit assessment. This calculation is tailored to the medication patterns of the target population under consideration. The daily intake of elements per kilogram of body mass is computed using the following formula:

Exp = (EF × Ed × IR × C)/(W × AT)(1)

Taking into account the medicinal and food properties of seaweeds, the exposure of elements was calculated in the following two situations: (1) For medicinal purposes: In Formula (1), Exp represents the exposure level of elemental content per kilogram of body mass per day, measured in μg/kg. EF denotes the exposure frequency of medicinal herbs, with the P_95_ of EF derived from the effective consumption survey, amounting to 90 days/year. Ed signifies the exposure duration over the lifetime of the medicinal herb, typically not exceeding 20 years. IR stands for the daily intake rate of medicinal herbs, measured in g/day, as per the dosage regulations outlined in the 2020 edition of the Chinese Pharmacopoeia. C denotes the residual amount of metallic elements in the herbs, measured in mg/kg. W represents the human body mass, based on statistical data from the National Health Commission, with the average weight of Chinese men and women in 2020 recorded as 69.6 kg and 59 kg, respectively. AT, as average lifespan in days, calculated at 70 years, amounts to 25,550 days. (2) Edible: According to survey data, the EF and exposure duration are 260 days/year; Ed represents the average lifespan of 70 years. IR denotes the daily intake rate of seaweed as food, with most regions incorporating seaweed into their diet as a vegetable; hence, this study sets IR at 30 g/day; all other parameters remain consistent with medicinal use. For elements participating in risk assessment, their risk levels are described by the Hazard Index (HI), calculated using the following formula:

HI = Exp × SF/PTDI(2)

Using Equation (2) to describe the risk characteristics of Pb, Cd, As, Hg, Cu, Al, Mn, Ni, Ca, Fe, Zn, Co, V, and Li in seaweeds and calculate the Hazard Index (HI). Where, when used as medicine, the safety factor (SF) is 10, and when used as food, the SF is 1; PTDI is the provisional tolerable daily intake of the element. The PTDI values for Pb, Cd, As, Cu, Al, Mn, and Ni are consistent with their health-based guidance values (HBGVs). According to the U.S. Environmental Protection Agency’s Integrated Risk Information System, we set the threshold for non-cancer risk at 1.0 [33]. If HI ≤ 1, the health risk is low and acceptable; if HI > 1, the risk is high and should be taken seriously.

#### 2.4.2. Carcinogenicity Risk Assessment

The first three steps of carcinogenic risk assessment align with those of non-carcinogenic risk assessment. The characterization of carcinogenic risk features represents the final step in carcinogenic risk assessment. In carcinogenic risk assessment, the characterization of carcinogenic risk features for Pb or As typically employs the carcinogenic risk (CR) [20]. CR refers to the probability of an individual developing cancer over their lifetime due to exposure to a specific heavy metal. The calculation formula for CR is as follows:

CR = Exp × CSF × 0.00(3)

In Equation (3), CSF stands for the carcinogenic slope factor (mg/kg/day)^−1^, indicating the probability risk of cancer from lifetime exposure to a specific carcinogen for a particular population. According to the technical report of the US EPA, the CSFs for Pb and As are 8.5 × 10^−3^ and 1.5 (mg/kg/day)^−1^, respectively. For assessment outcomes, the US EPA sets the acceptable risk levels for carcinogens within the range of 10^−6^ to 10^−4^ [33]. Generally, if CR < 10^−4^, the carcinogenic health risk of the element in TCMs and foods is considered acceptable; conversely, if CR > 10^−4^, heightened attention should be paid to the carcinogenic health risk of the element in TCMs and foods.

#### 2.4.3. Evaluation of Benefits

The core determined the Recommended Daily Intake (RDI) and Daily Reference Intake (DRI) for each element. According to the revised standards of the “Chinese Residents Dietary Nutrient Reference Intake (2023 Edition),” the Recommended Daily Intake (RDI) for Ca, K, Na, Mg, Fe, Zn, Cu, and Cr are 18,660.0, 55,990.0, 31,100.0, 5130.0, 310.0, 190.0, 10.0, and 470.0 μg/(kg·d), respectively. Additionally, the Daily Reference Intake (DRI) for Pb, Cd, As, and Hg are 0.3, 0.1, 0.2, and 0.3 μg/(kg·d), respectively; Thirdly, calculating exposure assessments using Formula (1) under the “non-carcinogenic risk assessment” category; Fourthly, the efficacy of the elements involved in the efficacy assessment is described by the Benefit Index (BI). The formula is as follows:

(4) BI=Exp×SF/RDI

In Equation (4), RDI stands for the Recommended Daily Intake of the element, measured in mg/(kg·d). If BI ≤ 1, the health benefit was considered low; if BI > 1, it was considered high.

#### 2.4.4. Construction of Risk–Benefit Assessment Models

The risk–benefit assessment model established based on the three species of seaweed integrates three evaluation factors: the Hazard Index (HI), Carcinogenic Risk (CR), and Benefit Index (BI) to comprehensively consider the health benefits and risks posed by specific elements to the human body. For a specific element, the assessment method is as follows. Initially, the results of the carcinogenic risk assessment are considered. If CR > 10^−4^, it is deemed that the carcinogenic health risk of the element should be prioritized, and non-carcinogenic health risks and health benefits should not be considered at this stage. If CR < 10^−4^, it is considered that the carcinogenic health risk of the element is acceptable, and the assessment continues for non-carcinogenic health risks and health benefits. Second, the assessment results of the Hazard Index (HI) and Benefit Index (BI) were considered together: if BI was <1, the health benefits were relatively low; if HI ≤ 1 ≤ BI, there is a risk–benefit balance; and if HI > 1, the health risk is higher.

## 3. Results

### 3.1. Multi-Element Analysis in Different Seaweeds

#### 3.1.1. Establishment of Elemental Fingerprints in Seaweeds

**Figure 1:**
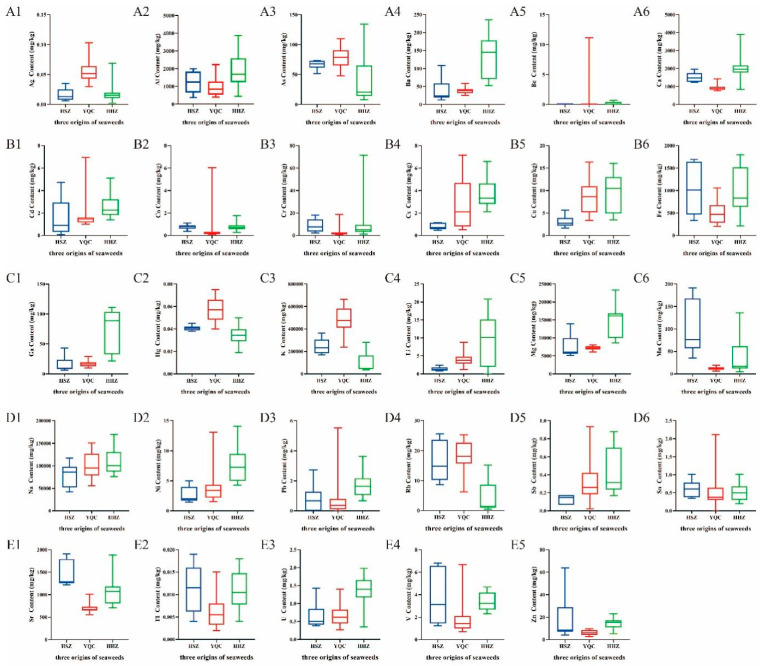
The mean values (mg/kg) of the 29 elements in seaweeds from different species. (**A1**–**A6**: Ag, Al, As, Ba, Be, and Ca; **B1**–**B6**: Cd, Co, Cr, Cs, Cu, and Fe; **C1**–**C6**: Ga, Hg, K, Li, Mg, and Mn; **D1**–**D6**: Na, Ni, Pb, Rb, Sb, and Sn; **E1**–**E5**: Sr, Tl, U, V, and Zn).

In this study, a total of 29 mineral elements were identified in seaweeds, and their fingerprints of the mineral elements of seaweeds were established. Table 2 and Figure 1 list the average content of the 29 mineral elements in the three seaweed species. Mineral elements were classified into three groups according to their average content in seaweeds: large amounts of elements (>50 mg/kg), low amounts of elements (1–50 mg/kg), and trace elements (<1 mg/kg).

#### 3.1.2. Study of Elemental Content in Seaweeds from Different Species

The large amounts of elements group included K, Na, Mg, Al, Ca, Sr, Fe, and As. As shown in Table 2, potassium (K) is the most abundant element, with an average content ranging from 96,004.32 mg/kg (HHZ) to 485,737.88 mg/kg (YQC), which acts as a plant growth regulator during the growth process of plants, dynamically adjusting the acid–base balance within plants by regulating osmotic pressure. Sodium (Na) content averages higher in YQC (100,741.27 mg/kg) and HHZ (109,218.39 mg/kg) compared to HSZ (79,907.74 mg/kg). The average contents of Mg, Al, and Ca in HHZ are the highest— 15,008.02 mg/kg, 1841.98 mg/kg, and 2041.55 mg/kg, respectively—of which calcium (Ca) is the most abundant mineral element in the human body and plays an important role in the development of human bones and the maintenance of normal physiological function of the heart [34]. The average content of Sr and Fe elements in HSZ is the highest, at 1453.24 mg/kg and 1031.64 mg/kg, respectively, while in YQC, their contents are the lowest. Iron (Fe) is an essential nutrient for the human body and plays a crucial role in the synthesis of hemoglobin and the transport of oxygen [35,36]. Arsenic (As) has relatively high average concentrations in YQC and HSZ, at 76.40 mg/kg and 66.41 mg/kg, respectively, but is lower in HHZ, averaging 40.84 mg/kg. Excessive intake of harmful heavy metals can lead to skin damage, cardiovascular diseases, and other health issues [37]. Previous research has indicated that seaweed plants can accumulate As, making excessive As levels in seaweeds a concern [38].

The low amount of elements group included Ba, Rb, Ga, Mn, Cu, Zn, Li, Ni, Cs, Cr, V, Cd, Sb, and Be. As shown in Table 2, the average contents of Ba, Rb, Ga, and Mn were all greater than 10 mg/kg, whereas the average contents of Cu and Zn were between 5–10 mg/kg, and the average contents of the remaining elements were less than 5 mg/kg. The average contents of Mn and Zn in HSZ were the highest, at 100.67 mg/kg and 18.43 mg/kg, respectively. The average contents of Ba and Ga elements in HHZ are the highest, at 131.24 mg/kg and 72.13 mg/kg, respectively. Sb and Be have the highest average contents in YQC, at 1.70 mg/kg and 1.48 mg/kg, respectively, and the differences in contents among the three species of seaweed are significant.

The trace element group included Pb, Co, U, Sn, Tl, Hg, and Ag. As shown in Table 2, the average contents of Pb, Co, and U elements are highest in HHZ, at 1.74 mg/kg, 0.78 mg/kg, and 1.35 mg/kg, respectively. The highest average content of Sn was in the HSZ (0.61 mg/kg). The highest average contents of Tl, Hg, and Ag were in the YQC, at 0.40 mg/kg, 0.06 mg/kg, and 0.06 mg/kg, respectively. Specifically, the average content of Tl in the YQC was 40 times that in the average contents in HHZ and HSZ, indicating a significant difference that warrants attention.

#### 3.1.3. Chemometric Analysis of Elemental Fingerprints

##### Two-Dimensional-Hierarchical Cluster Analysis (2D-HCA)

**Figure 2:**
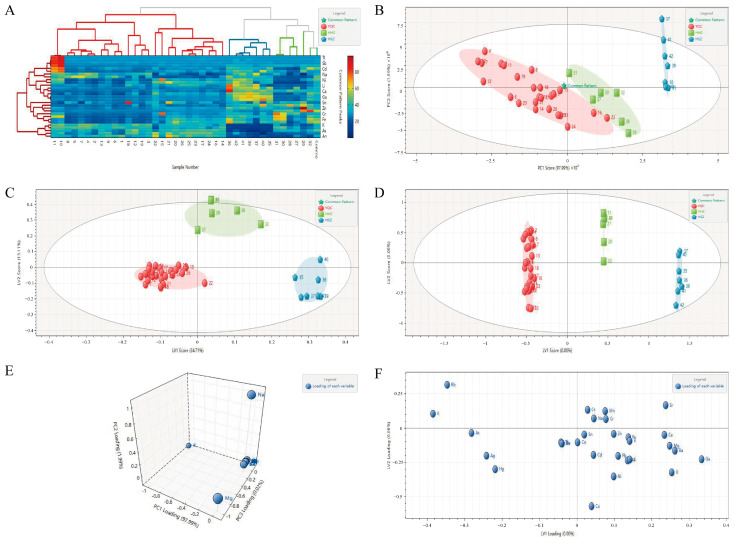
Dendrograms of the HCA (**A**); score plots of the PCA model (**B**); score plots of the PLS-DA model (**C**); score plots of the OPLS-DA model (**D**); loading plots of the PCA model (**E**); and loading plots of the OPLS-DA model (VIP > 1, *p* < 0.05) (**F**).

In this study, we applied the 2D-HCA algorithm to analyze the metal element content of seaweed samples from three different species. During the analysis, automatic scaling was employed for data preprocessing, and correlation coefficients and nearest neighbor methods were used to distinguish seaweed multi-element profiles (MEPs), as depicted in Figure 2A. Cluster analysis revealed that the 40 seaweed samples could be divided into four main clusters: red representing YQC, green representing HHZ, and blue representing HSZ. Three of the groups with significant deviations were excluded from HHZ. The YQC group was further divided into two clusters, whereas the HHZ and HSZ groups formed a single cluster. Therefore, the results of the 2D-HCA analysis indicated that MEPs from seaweeds of the same species were similar, whereas MEPs from seaweeds of different species exhibited significant differences. The findings of this study suggest that, based on the seaweed multi-element fingerprint, differences in elemental content among seaweeds from three species (YQC, HHZ, and HSZ) were successfully revealed, allowing for the discrimination of seaweeds from different species at the elemental level. However, HCA analysis alone cannot provide specific details regarding the correlation between mineral elements and seaweed samples. Further statistical analyses are required to clarify this relationship.

##### Principal Component Analysis (PCA)

To explore the feasibility of multi-element fingerprinting for seaweed species identification, principal component analysis (PCA) and partial least squares discriminant analysis (PLS-DA) were employed in this study. In this study, PCA was applied to analyze the mineral element data of all seaweed samples, yielding PCA score plots (Figure 2B). As shown in the score plot, the three species of seaweed samples (YQC, HHZ, and HSZ) were found to be distinctly differentiated. The cumulative contribution rate of the first three principal components, PC1, PC2 and PC3, was 100.0%, with PC1 explaining 97.99% of the cumulative variance (R_2_X). After conducting a validity test on the principal components at a significance level of α = 0.05, the null hypothesis (H_0_) was rejected, indicating statistically significant differences. Additionally, based on the seaweed multi-element loading plots (Figure 2E), detailed indicators causing differences among the different seaweed samples could be inferred. The loading plots show that the main characteristic indicators in the PC1, PC2, and PC3 directions are Na, Mg, Al, Ca, K, Sr, and Mn. These elements were identified as the characteristic mineral elements of different seaweeds and could therefore be utilized to infer specific details contributing to variations among seaweed species to a certain extent.

##### Partial Least Squares Discriminant Analysis (PLS-DA)

To achieve better classification and predictive models for the seaweed samples, we employed the PLS-DA model to analyze multivariate data, building on the foundation of the PCA model. The methodology for establishing the PLS-DA model was as follows: Data preprocessing involved the normalization of variables, retaining 10 latent variables, and employing k-fold cross-validation with k set to 10, which indicated 10 iterations for training subset extraction and validation. To enhance the reliability of cross-validation, we conducted 100 random simulations. A partial least squares regression model quality evaluation was conducted to assess the quality of the model. The model exhibited an X explained variance (R_2_X) of 0.929, a Y explained variance (R_2_Y) of 0.984, an X prediction rate (Q_2_X) of 0.953, and a Y prediction rate (Q_2_Y) of 0.944, indicating the strong explanatory and predictive capabilities of the model. As illustrated in Figure 2C, different seaweed samples from various species were well separated using this model. Specifically, the two species of seaweed samples, YQC and HHZ, exhibited significant differences, which is consistent with the classification results of the PCA model.

##### Orthogonal Partial Least Squares-Discriminant Analysis (OPLS-DA)

To achieve more accurate data extraction and classification, we employed the OPLS-DA model to analyze multi-element data from seaweed samples of different species. In this analysis, the following settings were applied: The number of retained latent variables was set to 29, and k-fold cross-validation was selected with k = 10, indicating that the training set was iteratively validated ten times. To enhance the reliability of cross-validation, the number of random simulations was set to 100. The results (Figure 2D) showed clear differentiation among seaweed samples from the YQC, HHZ, and HSZ, with significant discrimination. However, among all the HHZ samples, two species of samples labeled 27 and 28 showed a trend deviating from the overall data, suggesting the possibility of adulteration. Additionally, to filter out key indicators with significant differences from multi-element data, filtering criteria were set with a VIP greater than 1 and P less than 0.05 to identify key components in the classification (Figure 2F). Following these criteria, we identified 14 variables with high significance (VIP > 1, *p* < 0.05), namely Ag, Ba, Ca, Cu, Fe, Ga, Mn, Rb, Sr, V, Cs, K, Na, and Hg. It was evident that there were many significant variables under these conditions. Therefore, we adjusted the filtering criteria to VIP > 1.5, *p* < 0.05, resulting in the identification of four variables: Ag, Mn, Sr, and K. These four elements were determined to be key elements. In conclusion, the OPLS-DA model provides effective feature extraction, data classification, and prediction and plays a crucial role in the identification of seaweed species.

### 3.2. Modeling of Risk–Benefit Assessment Based on Three Species of Seaweeds

#### 3.2.1. Results of Non-Carcinogenicity Assessment

**Figure 3:**
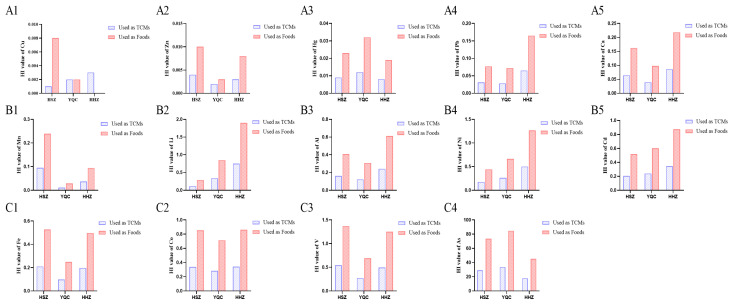
HI values of metal elements in seaweeds of different species used as TCMs and foods. (**A1**–**A5**: Cu, Zn, Hg, Pb, and Ca; **B1**–**B5**: Mn, Li, Al, Ni, and Cd; **C1**–**C4**: Fe, Co, V, and As).

The HI results of the relevant elements in the three seaweeds (Tables S1–S3, Figure 3) showed that for HSZ, the mean HI values of different metallic elements followed the sequence Cu < Zn < Hg < Pb < Ca < Mn < Li < Al < Ni < Cd < Fe < Co < V. For YQC, the mean HI values of different metallic elements followed the sequence Zn < Cu < Mn < Hg < Pb < Ca < Fe < Al < Cd < Ni < V < Co < Li < As; for HHZ, the mean HI values of different metallic elements followed the sequence Cu < Zn < Hg < Mn < Pb < Ca < Fe < Al < Co < Cd < V < Ni < Li < As. Based on these results, the differences in the mean HI values of the same metallic elements among the three seaweeds of different species indicate that there were significant variations in the non-carcinogenic risks. When seaweeds were used as medicines, the HI values of As in all batches of the three species of seaweed were significantly higher than 1, indicating a certain health risk associated with As in seaweeds, which should be taken seriously. As arsenic exists in various chemical forms and oxidation states, studies have shown that the toxicity of inorganic arsenic is much higher than that of organic arsenic, trivalent arsenic is more toxic than pentavalent arsenic, and arsenobetaine and arsenocholine are essentially non-toxic. Therefore, in addition to focusing on the total As content in seaweed, attention should be paid to its different chemical forms and oxidation states to conduct risk assessments in a more scientifically rational manner. In the HHZ samples, the HI values of Li were higher than 1 in all four batches, indicating the need for attention to the associated health risks. When seaweed is used as a food, health risks may be higher owing to longer exposure times, higher frequency of exposure, and greater consumption quantities.

#### 3.2.2. Results of Carcinogenicity Assessment

**Figure 4:**
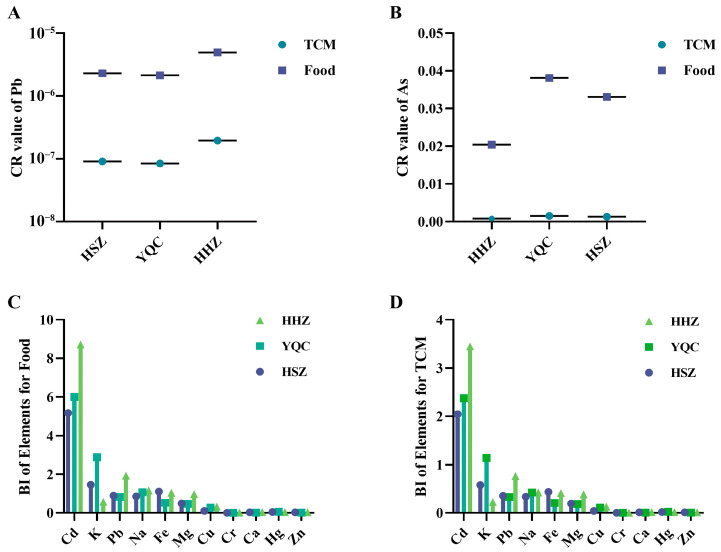
CR values of Pb and As in seaweeds of different species used as medicine and food (**A**,**B**). BI values of metal elements in seaweeds of different species used as medicine and food (**C**,**D**).

Previous studies have shown that the toxicity of elements is closely related to their forms and valence states. Based on the principle of protecting the majority of consumers, we assumed that the As form was the most toxic inorganic arsenic in all of the seaweed samples. The corresponding carcinogenicity risk (CR) of seaweeds for medicinal use and consumption was calculated using Equation (3). The results of the carcinogenicity assessment of Pb and As in the three species of seaweeds showed that (Table 3 and Figure 4A,B), regardless of whether the three species of seaweeds were used for medicinal use or for consumption, the CR of Pb in all batches of samples was lower than 1 × 10^−4^, indicating that the risks were acceptable; however, for As in seaweed samples, the CR for As was higher than 1 × 10^−4^ in all batches of samples, and therefore the health risk of As in seaweeds needs to be paid extra attention.

#### 3.2.3. Results of Benefits Evaluation

The BI results for the corresponding elements of the three species of seaweeds showed (Tables S4 and S5, Figure 4C,D) that when the seaweeds were used medicinally, the mean BI values of As and Cd were higher than 1 in the HSZ and HHZ samples, and the mean BI values of K, As, and Cd were higher than 1 in the YQC sample, suggesting higher health benefits, while the mean BI values of the remaining elements were lower than 1; when seaweeds were used as foods, the BI mean values of Fe, K, Cd, and As in HSZ samples were all higher than 1. In the YQC samples, the mean BI values of Na, K, Cd, and As were >1. In the HHZ samples, the mean BI values of Fe, Na, Pb, Cd, and As were all higher than 1, indicating their significant health benefits. These results indicate that for seaweeds of different species, the health benefits of the corresponding elements are different, and the health benefits of different consumption methods are also different. Special analyses should be conducted according to different application scenarios. In addition, when evaluating the benefits of the corresponding elements, it is necessary to combine the risk assessment results of these elements and consider their risks and benefits to obtain accurate and comprehensive evaluation results.

#### 3.2.4. Results of Modeling Risk-Benefit Assessment

First, according to the carcinogenic risk assessment results, the cancer risk (CR) of arsenic (As) in all batches of samples from the three species of seaweeds exceeded 1 × 10^−4^. This indicates that whether used as TCM and food, the ingestion of seaweed carries a certain level of carcinogenic risk, necessitating heightened attention to its health implications (Table 4). For Pb, the CR of Pb in all seaweed samples was less than 1 × 10^−4^, indicating an acceptable carcinogenic health risk for Pb in seaweeds. Second, based on the non-carcinogenic assessment results (HI) and benefit evaluation results (BI), for constant elements, the mean BI values of Ca in the three species of seaweeds were 0.011, 0.006, and 0.014, respectively, indicating that the health benefits of Ca in the three species of seaweeds were relatively low. The mean BI values of K were 0.576, 1.141, and 0.225, respectively, indicating that the health benefits of K in the YQC samples were relatively high. The mean BI values of Mg in the three species of seaweeds were 0.194, 0.185, and 0.385, respectively, whereas those of Na were 0.338, 0.426, and 0.462, respectively. These results indicated that the health benefits of Mg and Na were relatively low. For the essential elements, the mean BI values of Fe in the three species of seaweeds were 0.438, 0.206, and 0.412, respectively, and the mean HI values of Fe were 0.098, 0.209, and 0.197, respectively, indicating that the health benefits of Fe were low. The mean BI values of Zn were 0.013, 0.004, and 0.010, respectively, and the mean HI values of Zn were 0.001, 0.004, and 0.003, respectively, indicating that the health benefits of Zn were low. The mean BI values of Cu and Cr in the three species of seaweeds were lower than 1, indicating lower health benefits. For heavy metals, the BIs of Pb in three species of seaweeds (HSZ-4, YQC-10, and YQC-11) were all higher than 1, and the HIs were all lower than 1, so the risk–benefit level of Pb basically remained in equilibrium. Moreover, the BIs of Pb in the other seaweed samples were all lower than 1, with a lower health benefit. The BIs of Cd in three species of seaweeds were 2.045, 2.375, and 3.450, respectively, and the HI were 0.238, 0.205, and 0.345, so the risk–benefit of Cd was in equilibrium. The BIs of Hg were 0.018, 0.025, and 0.015, respectively, which indicated that it had a low health benefit. These findings highlight the need to address these issues.

## 4. Discussion

### 4.1. Chemometrics Analysis

This study focused on three different species of seaweed (YQC, HHZ, and HSZ); there is a certain degree of morphological similarity among these three species of seaweed. It is difficult to distinguish them solely based on morphological features, especially considering that most seaweed in circulation on the market is not in its original plant form but rather processed into segments or other forms. This significantly complicates the identification of their species. In this context, there is an urgent need for a method that is rapid, accurate, stable, widely applicable, and possesses strong discriminatory capabilities to reduce market confusion and alleviate public concerns. Therefore, fingerprint profiles of mineral elements were established for these species of seaweed. Chemical multivariate methods, including HCA, PCA, and PLS-DA, were employed for the analysis. The HCA results indicated that seaweeds of the same species clustered together, whereas those of different species showed discrete distributions. However, HCA lacked specificity in correlating mineral elements with seaweed samples. PCA results demonstrated distinct separation among the three species of seaweed, with the first three principal components explaining 100% of the variance, suggesting that underlying factors contributed to differences among species, although the optimal principal components were not confirmed. The PLS-DA results confirmed the improved classification and precise prediction of the seaweed samples under these conditions, consistent with the PCA findings. The OPLS-DA results underscored the significant differences among seaweed samples of different species. Cross-validation reliability was found to be enhanced through 100 random permutations, and the filtering criteria (VIP > 1.5, *p* < 0.05) identified four key factors: Ag, Mn, Sr, and K. Ag is typically found in trace amounts in seaweed. Our research indicates that the average concentrations of Ag in *Sargassum fusiforme* (*Harv.*) Setch. (YQC), *Sargassum pallidum* (*Turn.*) C.Ag. (HHZ), and *Sargassum miyabei* (HSZ) are 0.06 mg/kg, 0.02 mg/kg, and 0.02 mg/kg, respectively. These concentrations suggest that Ag levels can distinctly differentiate the three types of seaweed. Furthermore, previous studies have shown that Ag in seaweed can inhibit normal cellular functions by binding to proteins or enzymes within the seaweed cells, thereby suppressing their growth. Ag ions may disrupt the metabolic processes of seaweed by interacting with receptors or enzymes on the cell membrane [39,40]. Due to the different structures of seaweed from various origins, the mechanisms of Ag absorption and accumulation vary among different species of seaweed. Mn is an essential trace element in the metabolism of seaweed, playing a crucial role in the oxygen-releasing reactions of photosynthesis. The varying abilities of different seaweed species to absorb and accumulate manganese make it a potential marker for distinguishing between species [41,42,43]. In our study, the average concentrations of Mn in *Sargassum fusiforme* (*Harv.*) Setch. (YQC), *Sargassum pallidum* (*Turn.*) C.Ag. (HHZ), and *Sargassum miyabei* (HSZ) are 12.15 mg/kg, 39.69 mg/kg, and 100.67 mg/kg, respectively. These concentrations clearly show that Mn can significantly differentiate the three types of seaweed. Additionally, the OPLS-DA analysis results confirm that Mn plays a significant role in distinguishing among these seaweed species. Therefore, the variation in Mn concentration can serve as one of the indicators to differentiate between various species of seaweed. Sr is an element with chemical properties similar to Ca. In our study, the average contents of Sr in *Sargassum fusiforme* (*Harv.*) Setch. (YQC), *Sargassum pallidum* (*Turn.*) C.Ag. (HHZ), and *Sargassum miyabei* (HSZ) were 694.20 mg/kg, 1088.04 mg/kg, and 1453.24 mg/kg, respectively. The content variation indicates that Sr can significantly differentiate the three types of seaweed; Sr distribution is closely related to the concentration of Ca in seawater, and seaweeds absorb these elements to build their cell walls and skeletons [44,45,46,47]. Different species of seaweed may exhibit different characteristics in the absorption and accumulation of strontium, making it an important element for distinguishing between seaweed species. K is a highly significant element in seaweed, playing a crucial role in its growth, metabolism, and adaptability. Firstly, in our study, the average contents of K in *Sargassum fusiforme* (*Harv.*) Setch. (YQC), *Sargassum pallidum* (*Turn.*) C.Ag. (HHZ), and *Sargassum miyabei* (HSZ) were 485,737.88 mg/kg, 96,004.32 mg/kg, and 245,242.67 mg/kg, respectively. These concentrations clearly show that K can significantly differentiate the three types of seaweed. Secondly, K is a vital component of the intracellular fluid, participating in the regulation of osmotic pressure within seaweed cells. The absorption capacity for K may vary among different seaweed species, and variations in K content can reflect the ecological adaptability of seaweed. K is essential for photosynthesis in seaweed, as it is involved in the regulation of enzyme activity and the facilitation of energy transformation during the photosynthetic process. Additionally, K plays a role in cellular metabolism, including protein synthesis and sugar conversion [48,49,50,51]. Since different species of seaweed have varying requirements in these processes, their abilities to absorb and accumulate K may differ, which can serve as an indicator for species differentiation. By analyzing the content of K in seaweed, we can gain a better understanding of the ecological characteristics and growth mechanisms of seaweed species.

Regarding chemometric analysis, it does have certain inherent limitations, particularly its sensitivity to data variability and sample heterogeneity. Firstly, data variability can lead to model instability. During specific experimental processes, samples may introduce noise due to inconsistent operations or instrumental errors, and when sample data is not evenly distributed, it can cause the model to produce errors when facing data from different batches or under different conditions. To improve this situation, we can reduce data variability through data preprocessing methods such as standardization, noise reduction, and smoothing. Sample heterogeneity may prevent the model from accurately capturing the characteristics of all samples, thereby affecting the model’s generalization and stability, mainly manifested in insufficient sample representation or class imbalance. To improve this, we can increase sample diversity and employ cross-validation techniques during the analysis process.

### 4.2. The Potential Risks Associated with Heavy Metal Accumulation

Seaweed, as a marine plant often used in food, health supplements, and pharmaceuticals, has the ability to absorb nutrients and trace elements from water, including a specific propensity for accumulating arsenic and incorporating it into its own elemental composition. This includes various heavy metals such as Pb, Cd, As, Hg, and Cu. According to our research, the health risks associated with consuming seaweed are greater than those related to medicinal use, due to varying abilities of different seaweed species to absorb heavy metals, which may pose distinct health risks. For instance, arsenic in three types of seaweed may pose a certain carcinogenic risk, and long-term accumulation of heavy metals like As and Cd in the human body can lead to chronic health issues, such as liver and kidney damage and neurological disorders. Moreover, excessive heavy metals can also damage the ecological environment and disrupt the balance of marine ecosystems [52,53,54]. Therefore, the long-term accumulation of heavy metals poses risks to both human health and the ecological environment and should be a focus of concern. To reduce heavy metal contamination in the cultivation and processing of seaweed, several measures should be taken: Firstly, improve the management and control of heavy metal pollution by reasonably controlling the density of seaweed cultivation to avoid over-reliance on a single sea area and reduce the accumulation of water quality pollution. Secondly, select sea areas with lower pollution for seaweed cultivation, avoid using areas heavily impacted by industrial and agricultural pollution, and conduct regular environmental monitoring to ensure that the concentration of heavy metals in water does not exceed safety limits. Thirdly, improve processing techniques to ensure the use of pollution-free equipment and technology during the processing, drying, and storage of seaweed to prevent secondary contamination. Fourthly, establish a strict quality control system to regularly test for heavy metals in seaweed products and ensure they meet safety standards [55,56,57,58]. Fifthly, utilize biotechnologies, such as bioaccumulation and detoxification techniques, to reduce heavy metal content in seaweed. Finally, establish and enforce strict regulations and standards to ensure the safety of medicinal and edible seaweed use.

### 4.3. The Innovative Risk–Benefit Assessment

Based on the identification of three species of seaweeds, according to the characteristics and distribution of different elements and from both medicinal and edible perspectives, guided by industry risk assessment principles, an innovative risk–benefit assessment model was successfully constructed based on the different species of seaweeds. For carcinogenic risk assessment, the focus is primarily on Pb and As. Carcinogenic risk is typically represented by the CR, which refers to the probability of an individual developing cancer over their lifetime due to exposure to a specific heavy metal. The carcinogenic risk is generally estimated through “carcinogenic dose–response relationship” models and is related to factors such as the frequency of food exposure, duration of exposure, intake amount, residue levels of the metal element, average human body weight, and the CSF. Regarding the threshold for carcinogenic risk, it is determined based on the “Guidelines for Carcinogen Risk Assessment” proposed by the U.S. Environmental Protection Agency (U.S. EPA), which is a widely accepted method for assessing the carcinogenic health risks of hazardous substances internationally [33]. According to the “Guidelines for Carcinogen Risk Assessment,” the acceptable risk level for carcinogens is in the order of 10^−6^ to 10^−4^. Therefore, we set the health risk threshold for carcinogens at 10^−4^. If the CR is less than 10^−4^, it is considered that the carcinogenic health risk of the element in traditional Chinese medicine or food is acceptable. Conversely, if the CR is greater than 10^−4^, it is considered that the carcinogenic health risk of the element in traditional Chinese medicine or food requires focused attention. For non-carcinogenic risk assessment, we conducted a non-carcinogenic risk assessment for the first time on elements such as Pb, Cd, As, Hg, Cu, Al, Mn, Ni, Ca, Fe, Zn, Co, V, and Li in seaweed. The threshold for non-carcinogenic risk is set based on the U.S. Environmental Protection Agency’s Integrated Risk Information System (IRIS), which states that when the non-carcinogenic risk value (HI) is less than 1, it will not cause significant adverse non-carcinogenic health effects on the exposed population.

**Figure 5:**
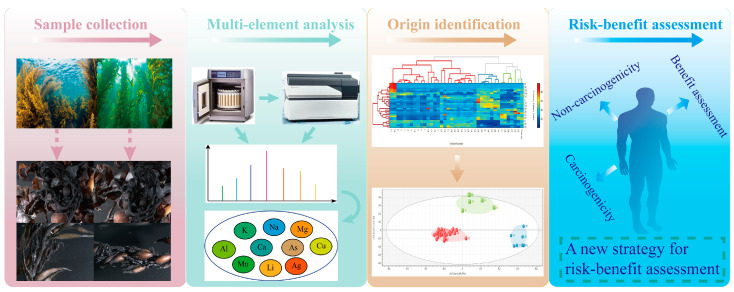
Schematic diagram of seaweeds in this study.

Regarding constant elements, when used medicinally, seaweed demonstrates higher health benefits for K, whereas when consumed, K, Na, and Mg exhibit higher health benefits in some samples, warranting attention. Regarding essential elements for the human body, the health benefits and risks of Fe, Zn, Cu, and Cr are relatively low. Concerning heavy metals and harmful elements, some samples of all three species of seaweeds had Pb risk–benefit balances, with lower health risks in the other samples. Moreover, most of the samples had balanced Cd health risks and benefits. For the first time in China, this model achieved a comprehensive assessment of the risk–benefit for three species of seaweeds through the integrated application of Exp, EF, CR, HI, and BI (Figure 5) It is hoped that this study can provide ideas for species identification and authenticity identification in both TCMs and foods and shed light on the establishment of a comprehensive assessment system for risk assessment and benefit evaluation of multi-elements in TCMs and foods, so as to provide a meaningful basis for reasonable and safe consumption by the public.

### 4.4. Suggestions for Regulatory Oversight and Quality Assurance in the Seaweed Industry

As a widely used ingredient in food and health products, the purity and quality of seaweed directly affect food safety and consumer health. However, due to the extensive sources and complex harvesting environments of seaweed, adulteration (such as the addition of non-ingredient components, low-quality seaweed, or other unsafe substances) occurs occasionally in some markets, which could lead to consumers ingesting harmful substances and even posing health risks. Additionally, consumers may be concerned about the presence of heavy metals and other contaminants in seaweed. For instance, according to our research findings, the levels of heavy metals in different types of seaweed can vary, with some heavy metals, such as total arsenic, being notably high. Our study provides some data and theoretical support regarding the safety concerns and functional applications of seaweed. Therefore, establishing effective quality control and regulatory frameworks is crucial. Based on our research results, to strengthen the regulation and quality assurance of the seaweed industry, we propose the following suggestions: (1) regulatory agencies could establish uniform testing standards using the chemometric methods we employed in our study, ensuring that the quality and purity of seaweed products on the market meet specified requirements; (2) enhance origin traceability, as seaweed samples from different origins may vary chemically, and establishing an origin traceability system could help regulatory agencies better track the source of seaweed, reducing the influx of substandard or adulterated seaweed into the market; and (3) increase regular quality inspections during the production, processing, and sales of seaweed, utilizing advanced instruments and chemometric analysis methods to identify and correct potential quality issues in a timely manner, ensuring the safety of seaweed products.

### 4.5. Considerations on the Socio-Economic Impact and Development Potential of Seaweed Within Circular Economy Frameworks

Seaweed, as a sustainable natural resource, is increasingly consumed globally due to its potential health benefits, such as being rich in nutrients, antioxidants, and dietary fiber. However, the socio-economic impact of seaweed consumption is not limited to health aspects; it also encompasses consumer behavior, industry development, economic benefits, and market dynamics. Our research provides a comprehensive assessment of the potential health risks and benefits associated with various types of seaweed. As consumer awareness of health issues grows, there is a tendency to opt for certified and quality-assured seaweed products. Our study offers scientific evidence and assurance to help consumers better discern high-quality seaweed products. Furthermore, seaweed also possesses considerable potential within the framework of a circular economy. Initially, the processing of seaweed generates certain waste materials, such as unused residual seaweed and effluents. If managed properly, these waste materials can be transformed into valuable resources under the circular economy model, serving as bioenergy, biofertilizers, and soil conditioners, among other uses. Additionally, seaweed plays a significant ecological role in aquaculture, including water purification, reduction of farming waste, and the establishment of green aquaculture systems. Furthermore, the integrated application of seaweed with aquaculture can provide a green production model for the industry, reducing reliance on external resources and mitigating the environmental impact during the farming process.

Additionally, in the actual processing and trade of seaweed, we preliminarily believe that implementing multi-element fingerprint analysis and risk–benefit assessment in this process is feasible from a cost–benefit perspective, as follows: Firstly, in terms of technical investment and costs, multi-element fingerprint analysis requires high-end equipment such as inductively coupled plasma mass spectrometry (ICP-MS) and laser-induced breakdown spectroscopy (LIBS). With technological advancements, an increasing number of related compact devices are also being developed, which necessitates the allocation of specialized laboratories and technical personnel. Secondly, regarding data processing costs, multi-element fingerprint analysis generates a large volume of data that requires professional software and data analysis teams for handling. Additionally, implementing risk assessments requires the development of relevant models and databases, which involves financial investment and technical maintenance. On the economic benefits side, multi-element fingerprint analysis can effectively ensure the quality of seaweed products, aiding producers in identifying and eliminating potential risks such as pollutants and heavy metals. This enhances product safety, strengthens consumer trust, and thereby improves market competitiveness and brand image. Secondly, as the international market demands increased food safety and environmental protection, many countries have strict testing standards for imported foods. Implementing multi-element fingerprint analysis and risk assessment helps enterprises meet these standards and increases opportunities to enter the international market. Thirdly, it can assist companies in better risk management and reduce losses. Fourthly, as seaweed is recognized as a sustainable natural resource, it is gaining more market attention. Implementing advanced detection and assessment technologies can help businesses align with sustainable development goals and further expand market opportunities [59,60,61]. In summary, implementing multi-element fingerprint analysis and risk assessment in seaweed processing and trade has a high degree of economic feasibility.

### 4.6. Analyze the PROSPECTS for Innovation in Industry 4.0

Currently, with the onset of Industry 4.0, the application of machine learning technology in multi-element analysis is burgeoning rapidly. For instance, Han and colleagues have suggested that the integration of elemental fingerprinting with machine learning modeling presents significant potential in the identification of edible animal blood products [62]. Nadai Fernandes and colleagues have successfully achieved traceability of Brazilian beef by combining elements from beef samples with machine learning algorithms, including multilayer perceptrons, random forests, and classification and regression trees [63]. Furthermore, AI-driven analytical innovations are showing robust vitality in the realms of traditional Chinese medicine and food. For example, Chung and colleagues utilized improved support vector machines based on scanned images of rice seeds to classify common pests and diseases [64]. Lucas Pires and colleagues applied support vector machines to achieve food quality control [65]. In summary, the application of machine learning and artificial intelligence technologies in traditional Chinese medicine and food is becoming increasingly widespread, enhancing the accuracy and scalability of predictions and offering broad developmental prospects.

## 5. Conclusions

In this study, based on the distinctive distribution characteristics of the elemental contents in three different seaweed species, pattern recognition algorithms (including supervised and unsupervised analyses) were employed to discriminate the species of the three seaweed species. Building upon this, innovative risk–benefit assessment models were proposed in accordance with guidance principles, allowing simultaneous consideration of risks and benefits for both food and TCM. A comprehensive, multi-element risk–benefit assessment of the three seaweed species was conducted scientifically and reasonably. Thanks for the reviewer’s comments. In the process of food safety regulation, the risk–benefit assessment model can be specifically applied through the following strategies: (1) establish a hierarchical structure model to clearly define the subject of evaluation; (2) create a judgment matrix to integrate data; (3) utilize the risk–benefit comprehensive evaluation method proposed in this study to conduct an integrated assessment of the research subject; (4) objectively optimize the weights; and (5) based on the results of the risk–benefit assessment, carry out risk management, capital allocation, and corporate optimization. We hope that this study will provide novel ideas for species identification and authenticity identification of TCMs and foods and provide insights into the risk assessment and safety standards of multi-elements in TCMs and foods, so as to enhance public awareness of rational consumption and improve public health and provide certain strategies to regulatory authorities for conducting scientific food safety risk–benefit assessments.

## Figures and Tables

**Table 1:** Sample collection information in the present study.

Species	Number of Samples	Location
*Sargassum fusiforme* (Harv.) *Setch*. (YQC)	26	ShanDong ZheJiang
*Sargassum pallidum* (Turn.) C.Ag. (HHZ)	10	ShanDong
*Sargassum miyabei*(HSZ)	6	GuangDong ShanDong

**Table 2:** The mean values (mg/kg) of the 29 elements in seaweeds from different species.

Elements	YQC (n = 26)		HHZ (n = 10)		HSZ (n = 6)	
	Mean	±SD	Mean	±SD	Mean	±SD
K	485,737.88	110,093.41	96,004.32	89,543.38	245,242.67	72,155.88
Na	100,741.27	26,994.16	109,218.39	29,994.79	79,907.74	26,980.11
Mg	7208.96	533.05	15,008.02	4486.02	7588.84	3339.17
Al	927.36	458.59	1841.98	983.48	1227.78	627.71
Ca	915.70	135.90	2041.55	768.55	1511.74	279.74
Sr	694.20	94.63	1088.04	342.08	1453.24	296.26
Fe	486.41	237.68	971.89	527.80	1031.64	606.18
As	76.40	16.11	40.84	40.84	66.41	7.92
Ba	38.45	7.15	131.24	62.26	38.93	35.38
Rb	18.74	4.50	4.36	5.55	16.27	6.85
Ga	16.82	4.25	72.13	35.03	15.59	14.02
Mn	12.15	3.30	39.69	43.27	100.67	60.84
Cu	8.25	3.42	9.62	4.35	3.07	1.37
Zn	6.45	2.21	14.70	5.07	18.43	22.70
Li	4.08	2.12	9.14	7.16	1.40	0.57
Ni	3.98	2.57	7.60	3.02	2.65	1.38
Cs	2.71	2.10	3.76	1.44	0.78	0.28
Cr	2.66	3.56	12.13	21.23	8.73	5.90
V	1.87	1.41	3.38	0.85	3.71	2.52
Cd	1.81	1.45	2.62	1.08	1.56	1.74
Sb	1.70	4.91	0.44	0.26	0.13	0.05
Be	1.48	3.66	0.31	0.29	0.04	0.02
Pb	0.78	1.35	1.74	0.86	1.22	1.02
Co	0.64	1.46	0.78	0.41	0.77	0.25
U	0.64	0.27	1.35	0.47	0.65	0.39
Sn	0.53	0.44	0.54	0.27	0.61	0.24
Tl	0.40	1.40	0.01	0.00	0.01	0.01
Hg	0.06	0.01	0.03	0.01	0.04	0.00
Ag	0.06	0.02	0.02	0.02	0.02	0.01

**Table 3:** CR values of Pb and As in different species of seaweeds used as TCM and food.

Batch No.	Pb		As		Batch No.	Pb		As		Batch No.	Pb		As	
	TCM	Food	TCM	Food		TCM	Food	TCM	Food		TCM	Food	TCM	Food
HSZ-1	7.82 × 10^−8^	1.98 × 10^−6^	1.35 × 10^−3^	3.40 × 10^−2^	YQC-9	2.91 × 10^−8^	7.35 × 10^−7^	1.21 × 10^−3^	3.06 × 10^−2^	YQC-24	6.89 × 10^−8^	1.74 × 10^−6^	1.81 × 10^−3^	4.59 × 10^−2^
HSZ-2	7.07 × 10^−8^	1.79 × 10^−6^	1.28 × 10^−3^	3.24 × 10^−2^	YQC-10	6.17 × 10^−7^	1.56 × 10^−5^	1.47 × 10^−3^	3.72 × 10^−2^	YQC-25	5.00 × 10^−8^	1.26 × 10^−6^	1.74 × 10^−3^	4.39 × 10^−2^
HSZ-3	8.99 × 10^−8^	2.27 × 10^−6^	1.45 × 10^−3^	3.67 × 10^−2^	YQC-11	5.30 × 10^−7^	1.34 × 10^−5^	1.48 × 10^−3^	3.73 × 10^−2^	YQC-26	3.42 × 10^−8^	8.65 × 10^−7^	1.81 × 10^−3^	4.58 × 10^−2^
HSZ-4	3.06 × 10^−7^	7.74 × 10^−6^	1.02 × 10^−3^	2.58 × 10^−2^	YQC-12	3.44 × 10^−9^	8.71 × 10^−8^	2.17 × 10^−3^	5.48 × 10^−2^	YQC-27	8.65 × 10^−8^	2.19 × 10^−6^	1.64 × 10^−3^	4.15 × 10^−2^
HSZ-5	NA	NA	1.33 × 10^−3^	3.36 × 10^−2^	YQC-13	1.28 × 10^−8^	3.25 × 10^−7^	1.52 × 10^−3^	3.83 × 10^−2^	HHZ-1	2.70 × 10^−7^	6.84 × 10^−6^	7.74 × 10^−4^	1.96 × 10^−2^
HSZ-6	NA	NA	1.43 × 10^−3^	3.62 × 10^−2^	YQC-14	4.73 × 10^−8^	1.20 × 10^−6^	1.64 × 10^−3^	4.16 × 10^−2^	HHZ-2	2.19 × 10^−7^	5.55 × 10^−6^	4.00 × 10^−4^	1.01 × 10^−2^
YQC-1	5.92 × 10^−9^	1.50 × 10^−7^	9.45 × 10^−4^	2.39 × 10^−2^	YQC-15	6.09 × 10^−8^	1.54 × 10^−6^	1.56 × 10^−3^	3.96 × 10^−2^	HHZ-3	4.06 × 10^−7^	1.03 × 10^−5^	3.01 × 10^−4^	7.61 × 10^−3^
YQC-2	0.00	0.00	9.44 × 10^−4^	2.39 × 10^−2^	YQC-16	9.03 × 10^−8^	2.28 × 10^−6^	1.83 × 10^−3^	4.63 × 10^−2^	HHZ-4	2.31 × 10^−7^	5.85 × 10^−6^	2.15 × 10^−4^	5.45 × 10^−3^
YQC-3	6.32 × 10^−8^	1.60 × 10^−6^	1.55 × 10^−3^	3.92 × 10^−2^	YQC-18	1.10 × 10^−7^	2.77 × 10^−6^	1.87 × 10^−3^	4.73 × 10^−2^	HHZ-5	1.51 × 10^−7^	3.82 × 10^−6^	2.64 × 10^−3^	6.68 × 10^−2^
YQC-4	1.19 × 10^−8^	3.02 × 10^−7^	1.38 × 10^−3^	3.49 × 10^−2^	YQC-19	2.88 × 10^−8^	7.29 × 10^−7^	1.78 × 10^−3^	4.49 × 10^−2^	HHZ-6	7.44 × 10^−8^	1.88 × 10^−6^	1.58 × 10^−4^	3.98 × 10^−3^
YQC-5	1.45 × 10^−9^	3.66 × 10^−8^	1.08 × 10^−3^	2.74 × 10^−2^	YQC-20	1.66 × 10^−8^	4.19 × 10^−7^	1.32 × 10^−3^	3.33 × 10^−2^	HHZ-7	1.67 × 10^−7^	4.22 × 10^−6^	4.14 × 10^−4^	1.05 × 10^−3^
YQC-6	8.95 × 10^−9^	2.26 × 10^−8^	1.06 × 10^−3^	2.69 × 10^−2^	YQC-21	1.22 × 10^−7^	3.09 × 10^−6^	1.81 × 10^−3^	4.58 × 10^−2^	HHZ-8	2.01 × 10^−7^	5.09 × 10^−6^	3.20 × 10^−4^	8.08 × 10^−3^
YQC-7	3.16 × 10^−8^	7.98 × 10^−7^	1.59 × 10^−3^	4.03 × 10^−2^	YQC-22	1.01 × 10^−7^	2.55 × 10^−6^	1.56 × 10^−3^	3.93 × 10^−2^	HHZ-9	1.22 × 10^−7^	3.08 × 10^−6^	1.15 × 10^−3^	2.90 × 10^−2^
YQC-8	NA	NA	1.35 × 10^−3^	3.42 × 10^−2^	YQC-23	5.90 × 10^−8^	1.49 × 10^−6^	1.04 × 10^−3^	2.64 × 10^−2^	HHZ-10	9.86 × 10^−8^	2.49 × 10^−6^	1.68 × 10^−3^	4.25 × 10^−2^

**Table 4:** The results of risk–benefit assessment based on three species of seaweeds used as TCM and food.

Elements	YQC				HHZ				HSZ			
	TCM		Food		TCM		Food		TCM		Food	
	CR	HI-BI	CR	HI-BI	CR	HI-BI	CR	HI-BI	CR	HI-BI	CR	HI-BI
As	>10^−4^	HI > 1	>10^−4^	HI > 1	>10^−4^	HI > 1	>10^−4^	HI > 1	>10^−4^	HI > 1	>10^−4^	HI > 1
Pb	<10^−4^	BI < 1	<10^−4^	BI < 1	<10^−4^	BI < 1	<10^−4^	HI < 1 < BI	<10^−4^	BI < 1	<10^−4^	BI < 1
V	/	HI < 1	/	HI < 1	/	HI < 1	/	HI > 1	/	HI < 1	/	HI > 1
Co	/	HI < 1	/	HI < 1	/	HI < 1	/	HI < 1	/	HI < 1	/	HI < 1
Fe	/	BI < 1	/	BI < 1	/	BI < 1	/	HI < 1 < BI	/	BI < 1	/	HI < 1 < BI
Cd	/	HI < 1 < BI	/	HI < 1 < BI	/	HI < 1 < BI	/	HI < 1 < BI	/	HI < 1 < BI	/	HI < 1 < BI
Ni	/	HI < 1	/	HI < 1	/	HI < 1	/	HI > 1	/	HI < 1	/	HI < 1
Al	/	HI < 1	/	HI < 1	/	HI < 1	/	HI < 1	/	HI < 1	/	HI < 1
Li	/	HI < 1	/	HI < 1	/	HI < 1	/	HI > 1	/	HI < 1	/	HI < 1
Mn	/	HI < 1	/	HI < 1	/	HI < 1	/	HI < 1	/	HI < 1	/	HI < 1
Ca	/	BI < 1	/	BI < 1	/	BI < 1	/	BI < 1	/	BI < 1	/	BI < 1
Hg	/	BI < 1	/	BI < 1	/	BI < 1	/	BI < 1	/	BI < 1	/	BI < 1
Zn	/	BI < 1	/	BI < 1	/	BI < 1	/	BI < 1	/	BI < 1	/	BI < 1
Cu	/	HI < 1 < BI	/	BI < 1	/	HI < 1 < BI	/	BI < 1	/	BI < 1	/	BI < 1
Na	/	BI < 1	/	BI > 1	/	BI < 1	/	BI > 1	/	BI < 1	/	BI < 1
K	/	HI < 1 < BI	/	BI > 1	/	BI < 1	/	BI < 1	/	BI < 1	/	BI > 1
Mg	/	BI < 1	/	BI < 1	/	BI < 1	/	BI < 1	/	BI < 1	/	BI < 1
Cr	/	BI < 1	/	BI < 1	/	BI < 1	/	BI < 1	/	BI < 1	/	BI < 1

**Notes:** The HI values for Na, Mg, and Cr in the three species of seaweed were not analyzed in this study. The BI values for V, Co, Ni, and Mn in the three species of seaweed were not analyzed in this study. The “/” indicates that the CR value was not analyzed for that particular element.

## Data Availability

The original contributions presented in this study are included in this article/Supplementary Material.

## Supplementary Materials

The following supporting information can be downloaded at: https://www.mdpi.com/article/10.3390/foods13244159/s1. Table S1: The mean of HI values of metal elements in seaweeds of different species used as medicine and food. Table S2: HI values of metal elements in seaweeds of different species used as medicine and food (Pb, Cd, As, Hg, Cu, Al, and Mn). Table S3: HI values of metal elements in seaweeds of different species used as medicine and food (Ni, Ca, Fe, Zn, Co, V, and Li). Table S4: The mean of BI values of metal elements in seaweeds of different species used as medicine and food. Table S5: BI values of metal elements in seaweeds of different species used as medicine and food (Ca, K, Na, Mg, Fe, and Zn).
