# Supplementary material for: Multi-Element Fingerprinting Combined with Chemometrics for Identification of Seaweeds and Innovative Risk–Benefit Assessment
Source: Foods. 2024 Dec 22;13(24):4159. doi: 10.3390/foods13244159 (PMC11675776; doi:10.3390/foods13244159)
Supplement: Supplementary file 1 [file foods-13-04159-s001.zip › Supplementary Material.pdf]

Supplementary Material

Table S1
The mean of HI values of metal elements in seaweeds of different species used as medicine and food.

Elements	HSZ		YQC		HHZ	
	TCM	Food	TCM	Food	TCM	Food
Cu	0.001	0.008	0.002	0.002	0.003	0.000
Zn	0.004	0.010	0.001	0.003	0.003	0.008
Hg	0.009	0.023	0.012	0.032	0.008	0.019
Pb	0.031	0.077	0.028	0.072	0.065	0.165
Ca	0.064	0.162	0.039	0.098	0.086	0.218
Mn	0.095	0.239	0.011	0.029	0.037	0.094
Li	0.115	0.291	0.336	0.849	0.751	1.899
Al	0.161	0.408	0.122	0.308	0.242	0.612
Ni	0.174	0.440	0.262	0.662	0.499	1.263
Cd	0.205	0.517	0.238	0.600	0.345	0.872
Fe	0.209	0.527	0.098	0.249	0.197	0.497
Co	0.338	0.854	0.282	0.713	0.340	0.861
V	0.543	1.372	0.273	0.690	0.494	1.249
As	29.106	73.573	33.484	84.641	17.899	45.245

Table S2

HI values of metal elements in seaweeds of different species used as medicine and food (Pb, Cd, As, Hg, Cu, Al, Mn).

batch No.	Pb		Cd		As		Hg		Cu		Al		Mn	
	TCM	Food	TCM	Food	TCM	Food	TCM	Food	TCM	Food	TCM	Food	TCM	Food
HSZ-1	0.026	0.066	0.622	1.573	29.921	75.634	0.010	0.025	0.001	0.011	0.262	0.662	0.179	0.453
HSZ-2	0.024	0.060	0.124	0.313	28.442	71.894	0.009	0.022	0.001	0.009	0.200	0.505	0.061	0.154
HSZ-3	0.030	0.076	0.111	0.279	32.228	81.465	0.008	0.021	0.001	0.007	0.231	0.584	0.065	0.164
HSZ-4	0.103	0.260	0.308	0.780	22.652	57.258	0.009	0.022	0.001	0.008	0.128	0.323	0.150	0.380
HSZ-5	0.000	0.000	0.054	0.137	29.547	74.689	0.009	0.023	0.001	0.006	0.049	0.125	0.078	0.198
HSZ-6	0.000	0.000	0.008	0.021	31.847	80.502	0.009	0.023	0.000	0.007	0.099	0.249	0.033	0.085
YQC-1	0.002	0.005	0.192	0.485	21.007	53.101	0.009	0.022	0.001	0.011	0.061	0.155	0.008	0.021
YQC-2	0.000	0.000	0.161	0.406	20.984	53.044	0.009	0.022	0.001	0.009	0.053	0.133	0.008	0.021
YQC-3	0.021	0.054	0.162	0.410	34.455	87.095	0.012	0.031	0.001	0.006	0.090	0.226	0.009	0.022

YQC-4	0.004	0.010	0.133	0.336	30.702	77.608	0.010	0.026	0.001	0.008	0.071	0.179	0.010	0.025
YQC-5	0.000	0.001	0.144	0.365	24.098	60.914	0.010	0.024	0.001	0.009	0.073	0.186	0.012	0.029
YQC-6	0.003	0.008	0.217	0.549	23.621	59.709	0.009	0.024	0.001	0.004	0.093	0.235	0.009	0.024
YQC-7	0.011	0.027	0.199	0.503	35.387	89.451	0.011	0.029	0.002	0.007	0.108	0.274	0.011	0.027
YQC-8	0.000	0.000	0.146	0.368	30.050	75.960	0.009	0.023	0.001	0.007	0.052	0.130	0.006	0.015
YQC-9	0.010	0.025	0.252	0.638	26.865	67.910	0.014	0.034	0.002	0.002	0.077	0.193	0.009	0.024
YQC-10	0.207	0.524	0.913	2.308	32.698	82.652	0.014	0.034	0.003	0.002	0.140	0.353	0.014	0.035
YQC-11	0.178	0.451	0.832	2.103	32.781	82.864	0.012	0.029	0.003	0.000	0.114	0.287	0.012	0.029
YQC-12	0.001	0.003	0.145	0.366	48.208	121.860	0.014	0.036	0.002	0.000	0.070	0.177	0.008	0.020
YQC-13	0.004	0.011	0.190	0.481	33.712	85.217	0.013	0.033	0.001	0.000	0.058	0.147	0.008	0.020
YQC-14	0.016	0.040	0.136	0.344	36.543	92.372	0.013	0.032	0.002	0.000	0.170	0.430	0.015	0.039
YQC-15	0.020	0.052	0.154	0.388	34.775	87.903	0.012	0.031	0.003	0.000	0.085	0.215	0.010	0.026
YQC-16	0.030	0.077	0.185	0.468	40.712	102.911	0.012	0.031	0.002	0.000	0.163	0.412	0.014	0.036

YQC-17	0.037	0.093	0.180	0.456	41.562	105.058	0.014	0.036	0.002	0.000	0.165	0.418	0.014	0.034
YQC-18	0.010	0.025	0.197	0.499	39.482	99.801	0.014	0.035	0.002	0.000	0.116	0.294	0.009	0.024
YQC-19	0.006	0.014	0.142	0.360	29.237	73.904	0.011	0.027	0.001	0.000	0.065	0.163	0.008	0.021
YQC-20	0.041	0.104	0.185	0.468	40.242	101.723	0.012	0.031	0.003	0.000	0.293	0.740	0.018	0.046
YQC-21	0.034	0.086	0.196	0.496	34.560	87.361	0.016	0.040	0.004	0.000	0.174	0.440	0.013	0.032
YQC-22	0.020	0.050	0.259	0.654	23.214	58.680	0.016	0.041	0.003	0.000	0.141	0.357	0.014	0.036
YQC-23	0.023	0.059	0.204	0.517	40.322	101.926	0.015	0.037	0.003	0.000	0.206	0.522	0.015	0.039
YQC-24	0.017	0.043	0.240	0.607	38.609	97.594	0.015	0.037	0.003	0.000	0.149	0.376	0.012	0.031
YQC-25	0.012	0.029	0.208	0.527	40.270	101.795	0.015	0.037	0.003	0.000	0.173	0.438	0.012	0.031
YQC-26	0.029	0.073	0.202	0.511	36.497	92.256	0.014	0.036	0.003	0.000	0.211	0.533	0.017	0.043
HHZ-1	0.091	0.230	0.377	0.953	17.207	43.496	0.011	0.028	0.004	0.000	0.230	0.581	0.016	0.039
HHZ-2	0.074	0.186	0.299	0.757	8.895	22.485	0.009	0.023	0.003	0.000	0.230	0.581	0.011	0.028
HHZ-3	0.136	0.345	0.224	0.566	6.693	16.917	0.004	0.010	0.002	0.000	0.214	0.540	0.016	0.040

HHZ-4	0.078	0.197	0.297	0.750	4.787	12.101	0.007	0.019	0.003	0.000	0.135	0.341	0.011	0.028
HHZ-5	0.051	0.129	0.670	1.695	58.750	148.507	0.008	0.021	0.003	0.000	0.509	1.286	0.128	0.323
HHZ-6	0.025	0.063	0.247	0.623	3.501	8.849	0.007	0.018	0.001	0.000	0.057	0.144	0.005	0.013
HHZ-7	0.056	0.142	0.408	1.032	9.199	23.253	0.008	0.021	0.003	0.000	0.368	0.930	0.037	0.092
HHZ-8	0.068	0.171	0.458	1.158	7.105	17.960	0.008	0.020	0.003	0.000	0.176	0.445	0.013	0.034
HHZ-9	0.041	0.104	0.286	0.724	25.519	64.506	0.006	0.016	0.001	0.000	0.175	0.442	0.089	0.225
HHZ-10	0.033	0.084	0.183	0.463	37.335	94.374	0.006	0.016	0.001	0.000	0.329	0.833	0.047	0.119

Table S3

HI values of metal elements in seaweeds of different species used as medicine and food (Ni, Ca, Fe, Zn, Co, V, Li).

batch No.	Ni		Ca		Fe		Zn		Co		V		Li	
	TCM	Food	TCM	Food	TCM	Food	TCM	Food	TCM	Food	TCM	Food	TCM	Food
HSZ-1	0.330	0.834	0.058	0.148	0.280	0.708	0.004	0.009	0.368	0.929	0.631	1.596	0.199	0.504
HSZ-2	0.129	0.326	0.053	0.135	0.343	0.867	0.002	0.004	0.352	0.891	0.996	2.517	0.096	0.243
HSZ-3	0.137	0.346	0.052	0.131	0.329	0.832	0.002	0.005	0.357	0.902	0.946	2.392	0.133	0.336
HSZ-4	0.238	0.602	0.066	0.167	0.103	0.260	0.014	0.034	0.487	1.231	0.222	0.561	0.082	0.207
HSZ-5	0.116	0.292	0.083	0.210	0.068	0.171	0.002	0.004	0.308	0.779	0.180	0.454	0.068	0.173
HSZ-6	0.095	0.241	0.070	0.178	0.129	0.326	0.001	0.002	0.156	0.393	0.281	0.709	0.112	0.283
YQC-1	0.106	0.268	0.035	0.088	0.043	0.108	0.001	0.002	0.041	0.103	0.113	0.285	0.099	0.251
YQC-2	0.100	0.253	0.035	0.088	0.041	0.105	0.001	0.002	0.041	0.105	0.106	0.269	0.140	0.355
YQC-3	0.139	0.353	0.038	0.096	0.065	0.164	0.001	0.003	0.060	0.151	0.166	0.420	0.145	0.366

YQC-4	0.148	0.374	0.036	0.090	0.059	0.150	0.001	0.002	0.057	0.145	0.134	0.339	0.220	0.556
YQC-5	0.120	0.302	0.036	0.091	0.055	0.138	0.001	0.002	0.064	0.161	0.147	0.372	0.330	0.835
YQC-6	0.114	0.288	0.032	0.082	0.068	0.172	0.001	0.002	0.068	0.171	0.167	0.422	0.126	0.318
YQC-7	0.211	0.534	0.038	0.095	0.081	0.206	0.001	0.003	0.077	0.194	0.195	0.493	0.241	0.610
YQC-8	0.118	0.299	0.034	0.086	0.041	0.104	0.001	0.002	0.051	0.130	0.104	0.262	0.306	0.774
YQC-9	0.215	0.543	0.041	0.105	0.061	0.154	0.002	0.004	0.060	0.151	0.164	0.414	0.275	0.695
YQC-10	0.570	1.440	0.036	0.092	0.118	0.297	0.002	0.005	2.649	6.696	0.976	2.467	0.238	0.602
YQC-11	0.504	1.274	0.036	0.091	0.101	0.256	0.002	0.005	2.236	5.652	0.833	2.105	0.265	0.671
YQC-12	0.261	0.659	0.045	0.114	0.058	0.147	0.001	0.003	0.063	0.160	0.180	0.455	0.317	0.800
YQC-13	0.147	0.371	0.038	0.097	0.048	0.122	0.001	0.002	0.049	0.124	0.135	0.341	0.311	0.786
YQC-14	0.233	0.588	0.033	0.083	0.144	0.365	0.001	0.003	0.135	0.341	0.290	0.732	0.315	0.795
YQC-15	0.860	2.174	0.043	0.110	0.092	0.231	0.002	0.004	0.193	0.488	0.211	0.533	0.248	0.627
YQC-16	0.266	0.671	0.040	0.101	0.135	0.342	0.002	0.004	0.139	0.351	0.298	0.754	0.234	0.590

YQC-17	0.207	0.523	0.035	0.088	0.142	0.359	0.002	0.005	0.135	0.342	0.288	0.728	0.356	0.900
YQC-18	0.203	0.514	0.036	0.090	0.099	0.251	0.001	0.003	0.093	0.236	0.201	0.507	0.701	1.771
YQC-19	0.149	0.376	0.034	0.085	0.051	0.129	0.001	0.002	0.056	0.142	0.125	0.316	0.348	0.879
YQC-20	0.357	0.902	0.037	0.093	0.213	0.539	0.002	0.005	0.220	0.555	0.433	1.095	0.431	1.089
YQC-21	0.269	0.680	0.038	0.096	0.157	0.396	0.002	0.005	0.141	0.357	0.334	0.845	0.380	0.962
YQC-22	0.370	0.935	0.060	0.153	0.107	0.271	0.002	0.005	0.121	0.306	0.290	0.734	0.425	1.074
YQC-23	0.280	0.708	0.042	0.106	0.156	0.394	0.002	0.005	0.160	0.404	0.338	0.855	0.643	1.624
YQC-24	0.305	0.770	0.044	0.111	0.113	0.285	0.002	0.004	0.129	0.327	0.258	0.652	0.237	0.600
YQC-25	0.279	0.704	0.041	0.104	0.122	0.308	0.001	0.003	0.129	0.326	0.274	0.692	0.672	1.699
YQC-26	0.278	0.702	0.044	0.111	0.188	0.475	0.002	0.005	0.162	0.410	0.341	0.862	0.725	1.832
HHZ-1	0.569	1.438	0.081	0.206	0.180	0.455	0.003	0.009	0.322	0.815	0.513	1.297	1.190	3.009
HHZ-2	0.614	1.552	0.085	0.215	0.175	0.443	0.004	0.011	0.291	0.735	0.610	1.543	1.009	2.550
HHZ-3	0.334	0.844	0.093	0.235	0.152	0.385	0.003	0.007	0.229	0.579	0.424	1.071	0.166	0.420

HHZ-4	0.502	1.269	0.092	0.233	0.107	0.271	0.003	0.009	0.254	0.642	0.428	1.083	0.864	2.184
HHZ-5	0.924	2.335	0.087	0.220	0.363	0.917	0.003	0.009	0.783	1.980	0.687	1.737	0.803	2.029
HHZ-6	0.283	0.714	0.081	0.205	0.042	0.106	0.001	0.003	0.114	0.289	0.341	0.863	1.378	3.483
HHZ-7	0.448	1.131	0.165	0.418	0.289	0.730	0.005	0.012	0.452	1.142	0.571	1.444	1.711	4.324
HHZ-8	0.355	0.896	0.079	0.201	0.135	0.342	0.003	0.008	0.252	0.636	0.335	0.846	0.000	0.000
HHZ-9	0.313	0.791	0.063	0.160	0.162	0.409	0.002	0.006	0.332	0.839	0.407	1.029	0.152	0.384
HHZ-10	0.654	1.654	0.036	0.090	0.360	0.911	0.002	0.005	0.376	0.951	0.623	1.576	0.238	0.601

Table S4

The mean of BI values of metal elements in seaweeds of different species used as medicine and food.

Elements	HSZ		YQC		HHZ	
	TCM	Food	TCM	Food	TCM	Food
Cr	0.002	0.006	0.001	0.002	0.003	0.009
Ca	0.011	0.027	0.006	0.016	0.014	0.036
Zn	0.013	0.032	0.004	0.011	0.010	0.026
Hg	0.018	0.045	0.025	0.063	0.015	0.038
Cu	0.040	0.102	0.108	0.274	0.127	0.320
Mg	0.194	0.492	0.185	0.467	0.385	0.972
Na	0.338	0.854	0.426	1.077	0.462	1.167
Pb	0.356	0.900	0.330	0.835	0.762	1.925
Fe	0.438	1.106	0.206	0.521	0.412	1.042
K	0.576	1.456	1.141	2.883	0.225	0.570
Cd	2.045	5.171	2.375	6.004	3.450	8.721
As	43.659	110.360	50.227	126.962	26.849	67.867

Table S5

BI values of metal elements in seaweeds of different species used as medicine and food (Ca, K, Na, Mg, Fe, Zn).

batch No.	Ca		K		Na		Mg		Fe		Zn	
	TCM	Food	TCM	Food	TCM	Food	TCM	Food	TCM	Food	TCM	Food
HSZ-1	0.010	0.025	0.484	1.223	0.379	0.957	0.220	0.555	0.588	1.485	0.012	0.030
HSZ-2	0.009	0.023	0.852	2.154	0.495	1.251	0.151	0.382	0.719	1.818	0.006	0.014
HSZ-3	0.009	0.022	0.608	1.536	0.386	0.976	0.158	0.401	0.690	1.745	0.006	0.016
HSZ-4	0.011	0.028	0.396	1.000	0.178	0.449	0.132	0.334	0.216	0.545	0.044	0.112
HSZ-5	0.014	0.035	0.447	1.130	0.236	0.596	0.148	0.373	0.142	0.358	0.006	0.014
HSZ-6	0.012	0.030	0.669	1.692	0.354	0.894	0.358	0.905	0.271	0.684	0.003	0.007
YQC-1	0.006	0.015	0.992	2.508	0.392	0.991	0.176	0.444	0.090	0.227	0.003	0.007
YQC-2	0.006	0.015	1.380	3.488	0.570	1.441	0.170	0.430	0.087	0.220	0.002	0.006
YQC-3	0.006	0.016	1.379	3.485	0.571	1.443	0.164	0.413	0.136	0.344	0.004	0.010

YQC-4	0.006	0.015	1.506	3.808	0.639	1.615	0.191	0.483	0.124	0.314	0.003	0.008
YQC-5	0.006	0.015	1.129	2.855	0.439	1.111	0.172	0.436	0.114	0.289	0.003	0.007
YQC-6	0.005	0.014	1.310	3.311	0.403	1.020	0.180	0.455	0.143	0.360	0.002	0.005
YQC-7	0.006	0.016	1.531	3.871	0.583	1.474	0.199	0.502	0.171	0.431	0.004	0.010
YQC-8	0.006	0.014	1.143	2.890	0.527	1.333	0.195	0.493	0.086	0.218	0.003	0.007
YQC-9	0.007	0.017	0.974	2.463	0.401	1.012	0.176	0.445	0.127	0.322	0.006	0.014
YQC-10	0.006	0.015	1.561	3.945	0.592	1.497	0.204	0.515	0.247	0.623	0.006	0.015
YQC-11	0.006	0.015	1.360	3.437	0.561	1.418	0.203	0.513	0.212	0.536	0.007	0.017
YQC-12	0.007	0.019	1.521	3.844	0.492	1.244	0.175	0.442	0.122	0.308	0.004	0.010
YQC-13	0.006	0.016	1.087	2.749	0.391	0.988	0.188	0.476	0.101	0.256	0.003	0.008
YQC-14	0.005	0.014	1.116	2.822	0.333	0.842	0.190	0.481	0.303	0.765	0.004	0.011
YQC-15	0.007	0.018	0.925	2.339	0.416	1.053	0.157	0.396	0.192	0.485	0.005	0.014
YQC-16	0.007	0.017	0.664	1.679	0.290	0.734	0.180	0.455	0.283	0.717	0.005	0.014

YQC-17	0.006	0.015	1.072	2.711	0.387	0.978	0.170	0.430	0.298	0.752	0.006	0.016
YQC-18	0.006	0.015	1.078	2.726	0.437	1.104	0.180	0.456	0.208	0.527	0.003	0.009
YQC-19	0.006	0.014	1.256	3.176	0.500	1.263	0.199	0.502	0.107	0.270	0.002	0.006
YQC-20	0.006	0.015	1.247	3.151	0.371	0.937	0.193	0.487	0.447	1.131	0.006	0.015
YQC-21	0.006	0.016	1.116	2.821	0.368	0.930	0.179	0.452	0.329	0.831	0.007	0.017
YQC-22	0.010	0.025	0.561	1.419	0.260	0.658	0.207	0.522	0.225	0.568	0.006	0.016
YQC-23	0.007	0.018	0.925	2.338	0.292	0.739	0.196	0.494	0.327	0.826	0.006	0.015
YQC-24	0.007	0.018	0.868	2.195	0.234	0.592	0.168	0.426	0.236	0.597	0.005	0.013
YQC-25	0.007	0.017	0.941	2.378	0.295	0.745	0.200	0.506	0.256	0.646	0.004	0.011
YQC-26	0.007	0.019	1.011	2.557	0.328	0.829	0.192	0.486	0.394	0.996	0.006	0.015
HHZ-1	0.014	0.034	0.106	0.268	0.533	1.348	0.391	0.987	0.377	0.954	0.011	0.028
HHZ-2	0.014	0.036	0.115	0.292	0.613	1.548	0.423	1.069	0.367	0.929	0.014	0.035
HHZ-3	0.015	0.039	0.102	0.258	0.384	0.972	0.259	0.654	0.319	0.807	0.010	0.024

HHZ-4	0.015	0.039	0.086	0.216	0.484	1.224	0.423	1.068	0.225	0.569	0.011	0.029
HHZ-5	0.015	0.037	0.663	1.676	0.338	0.854	0.407	1.029	0.761	1.923	0.011	0.029
HHZ-6	0.014	0.034	0.127	0.320	0.715	1.808	0.461	1.165	0.088	0.222	0.004	0.009
HHZ-7	0.028	0.070	0.107	0.269	0.403	1.018	0.599	1.515	0.606	1.531	0.016	0.040
HHZ-8	0.013	0.033	0.084	0.211	0.377	0.954	0.419	1.060	0.283	0.716	0.010	0.026
HHZ-9	0.011	0.027	0.345	0.872	0.321	0.810	0.245	0.618	0.339	0.858	0.008	0.020
HHZ-10	0.006	0.015	0.520	1.315	0.450	1.136	0.220	0.557	0.756	1.910	0.006	0.016
